# Establishing a New Method of Artificial Respiration in Asphyxia Neonatorum

**Published:** 1894-03

**Authors:** 


### BOOK NOTICES.

Establishing a New Method of Artificial Respiration in Asphyxia Neonatorum. By J. Harvie Dew, M. D. New York: Press of Stettiner Lambert & Co., 22, 24, and 26 Reade Street. 1893.

This work is a pamphlet of sixteen pages devoted to the exposition of a new method of overcoming asphyxia in new-born children, with photographic illustrations.

The directions are: " To grasp the infant with the left hand, allowing the neck to rest between the thumb and forefinger, the head falling far over backward, straightening the mouth with the larynx and trachea, thereby serving to raise and hold open the epiglottis. The upper portion of the back and scapulæ resting in the palm of the hand, the other three fingers to be inserted in the axilla of the baby's left arm, raising it upward and outward. Then with the right hand, if the baby is large and heavy, grasp the knees in such a way as to hold them with the right knee resting between the thumb and forefinger, the left between the fore and middle fingers. This position will allow the back of the thighs to rest in the palm of the operator's hand. If the infant is small and light, it will be found more convenient and easier to hold it in the same way by the ankles instead of the knees, allowing the calves instead of the thighs to rest in the palm of the hand. The next step is to depress the pelvis and lower extremities, so as to allow the abdominal organs to drag the diaphram downward, and with the left hand to gently bend the dorsal region of the spine backward. This enlarges the thoracic cavity and produces inspiration. Then to excite expiration, reverse the movement, bringing the head, shoulders, and chest forward, closing the ribs upon each other, and at the same moment bring forward the thighs, resting them upon the abdomen. This movement arches the lumbar region backward, and so bends the child upon itself as to crowd together the contents of the thoracic and abdominal cavities, resulting in a most complete and forcible expiration. While this movement is a powerful one, the operator can, by his manipulations, accomplish it without shock and render it as gentle as he pleases."

The above method seems to the editor so rational, so effective, and so simple that he is impelled to give it verbatim from the author's pamphlet that as many of our readers may learn it as possible. In the original pamphlet it is illustrated by photographic views that make the explanation more clear.

The paper was read before the New York Academy of Medicine February 2d, 1893.

